# Surgical clip migration following laparoscopic cholecystectomy: A rare cause of acute cholangitis

**DOI:** 10.1016/j.amsu.2020.08.052

**Published:** 2020-09-08

**Authors:** Abdelkader Mizouni, Houssem Ammar, Mohamed Amine Said, Fathia Harrabi, Waad Farhat, Linda Ghabry, Rahul Gupta, Mohamed Ben Mabrouk, Ali Ben Ali

**Affiliations:** a University of Sousse, Department of Gastrointestinal Surgery, Sahloul Hospital, Sousse, Tunisia; b Department of Gastrointestinal Surgery, Synergy Institute of Medical Sciences, Dehradun, India

**Keywords:** Laparoscopic cholecystectomy, Surgical clip migration, Cholangitis

## Abstract

Clip migration following laparoscopic cholecystectomy (LC) is a rare and late complication of LC. The first case of surgical clip migration after LC was reported in 1992, and since then less than 100 cases have been reported in the literature. We report the case of cholangitis secondary to a surgical clip migration in an 83 years old male patient, 8 years after LC. Contrast-enhanced computed tomography of the abdomen (CT) showed intra and extrahepatic ducts dilatation secondary to a hyperdense object located in the distal common bile duct (CBD).

It was removed successfully from the CBD by endoscopic retrograde cholangiopancreatography after sphincterotomy. At the last follow-up of one year after her admission, the patient is symptom-free with normal liver enzyme and abdominal CT. Surgical clip migration into CBD, should be included in the differential diagnosis while treating patients with the past surgical history of LC. Early diagnosis and treatment of this complication can avoid serious complications.

## Highlights

• Clip migration following laparoscopic cholecystectomy (LC) is a rare and late complication of LC.
• Surgical clip migration into CBD following LC remains not well known by the surgeon because of its rarity.
• Early diagnosis can lead to a better prognostic.
• The exact physiopathological process of clip migration still not clear.

## 1 Introduction

Laparoscopic Cholecystectomy (LC) represents the gold standard for the treatment of gallstone disease [1]. The most common surgery-associated complications are bile duct injury and bleeding [2]. Surgical clip migration into the common bile duct (CBD) following LC is rare [[1], [2], [3]]. The exact pathophysiological process of clip migration is still not clear. It can cause serious complications especially when it is misdiagnosed [2]. Therefore an early diagnosis can avoid life-threatening complications and lead to a better prognosis. We present a case of cholangitis caused by a migrated surgical clip into CBD eight years after surgery. This case has been reported in line with the SCARE criteria [4].

### 1.1 Case description

**Fig. 1:**
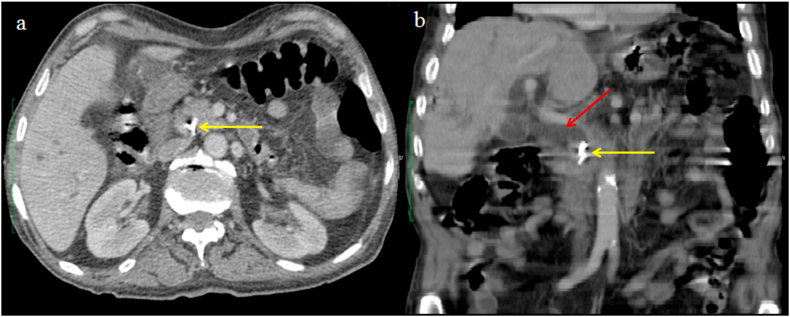
Contrast-enhanced computed tomography showing a surgical clip in the distal CBD (yellow arrow) associated with widened CBD up to 1,5 cm (red arrow) on axial (a) and coronal (b) sections. (For interpretation of the references to colour in this figure legend, the reader is referred to the Web version of this article.)

**Fig. 2:**
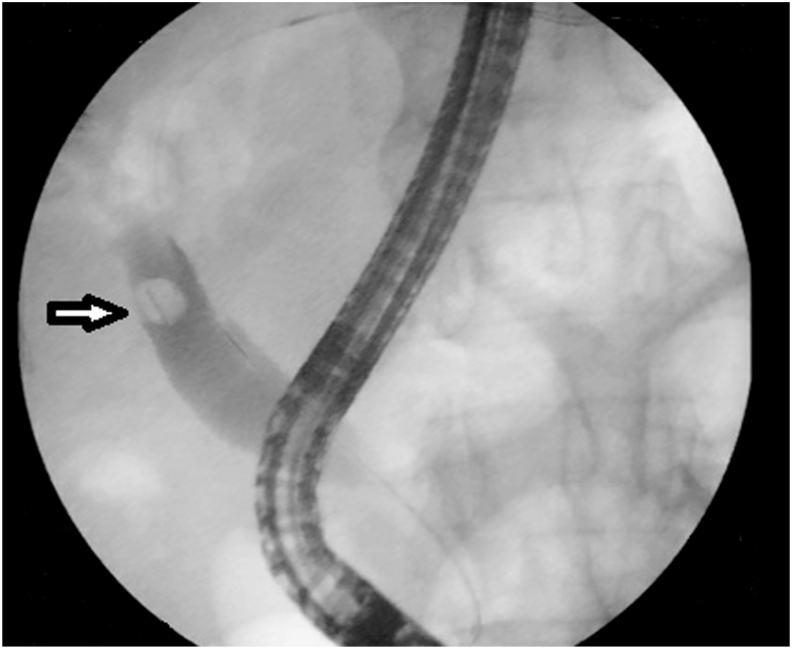
ERCP showing intra and extra ductal dilatation secondary to an occluding stone in the CBD formed around surgical clip (white arrow).

An 83-year-old man, known case of diabetes mellitus taking insulin, presented with severe abdominal pain, nausea, and jaundice for 3 days. Past surgical history included uneventful laparoscopic cholecystectomy for cholelithiasis 8 years back, with intraoperative cholangiogram showing clear bile ducts. The patient has episodic right upper quadrant abdominal pain for the past six months. The abdominal pain lasted for less than 6 hours without jaundice or fever. However, he has developed jaundice since three days. On physical examination, he was febrile (temperature of 38.5 °Celsius), had tachycardia, icterus, and tenderness in the right upper quadrant. Laboratory investigations revealed leucocytosis and elevated liver enzymes: alanine aminotransferase (ALT)260 U/L (N 7–40), aspartate aminotransferase (AST) 240 U/L (N 7–40), gamma-glutamyl transferase (GGT) 330 U/L (N 7–32) and a total bilirubin level of 102 μmol/L (N 5.1–17) with a direct bilirubin level of 91 μmol/L. Contrast-enhanced computed tomography of the abdomen (CT) showed intra and extrahepatic ducts dilatation secondary to a hyperdense object located in the distal common bile duct (CBD) as shown in Fig. 1. Based on clinical symptoms, laboratory results, and tomography findings, the diagnosis of cholangitis was confirmed. Broad-spectrum antibiotics and fluid resuscitation were started immediately and an urgent endoscopic retrograde cholangiopancreatography (ERCP) was performed. The ERCP revealed intra and extra ductal dilatation secondary to an occluding stone in the CBD formed around the surgical clip (Fig. 2). An endoscopic sphincterotomy was performed and the clip was removed. The patient was discharged after 6 days of hospitalization. At the last follow-up of one year after his admission, the patient is symptom-free with normal liver enzymes and abdominal CT.

## 2 Discussion

Clip migration into CBD is a rare and late complication following LC. The first case of surgical clip migration after LC was reported in 1992 [5]. The metallic clips migrated into the CBD act as a nidus for stone formation [2]. The common clinical symptoms of clip migration are right upper abdominal pain (approximately 70%–80% of cases), jaundice (approximately 50%–75%), and fever (approximately 30%) [3,6]. The time of clip migration can vary from 11 days to 20 years, while the median time of migration is usually 2 years and the median number of the migrated clip is 1 (range 1–6) [3]. The exact mechanism that leads to the migration of the surgical clip remains unknown. Kitamura et al. suggested that compression of the clipped cystic duct (CD) stump by adjacent structures especially liver, can be the cause of surgical clip migration into the lumen of CBD [2]. Furthermore, it is thought that incomplete closure of CD, the formation of bilioma, placement of too many clips or necrosis of clipped CD stump may lead to the migration of surgical clip into the CBD [3,7]. Also, the localized inflammation around the clips can also lead to erosion of the adjacent structures such as CBD and duodenum [7]. Hence, some authors have suggested the use of absorbable sutures, minimum number of clips and the accurate clip placement away from the CD and CBD junction to prevent clip migration [3,8]. ERCP is the first approach for treating clip migration, with a success rate of almost 85%, while surgery should be reserved for unsuccessful procedures [2,5]. Clip migration after cholecystectomy can also lead to other gastrointestinal complications, such as duodenal ulcer and pancreatitis [9].

In conclusion, surgical clip migration into CBD is a rarely encountered complication following LC. It should be included in the differential diagnosis while treating patients with the past surgical history of LC. Early diagnosis and treatment of this complication can avoid serious complications.

## Informed consent

The patient provided informed written consent prior to submission of this manuscript.

## Ethical approval

The study was approved by Ethics Committee.

## Source of funding

This study has not received any funding.

## Author contributions

Study concept or design – MBM, HA, Data collection – HA, WF, RG. Data interpretation – MBM, AM, LG. Literature review – WF, FH, LG,ABA. . Editing of the paper – MBM, MAS,AM.

## Trial registry number

1. Name of the registry:
2. Unique Identifying number or registration ID:
3. Hyperlink to the registration (must be publicly accessible)

## Guarantor

Houssem Ammar.

## Declaration of competing interest

The authors declare no conflict of interest.
